# Identification and characterization of unrecognized viruses in stool samples of non-polio acute flaccid paralysis children by simplified VIDISCA

**DOI:** 10.1186/1743-422X-11-146

**Published:** 2014-08-12

**Authors:** Shahzad Shaukat, Mehar Angez, Muhammad Masroor Alam, Maarten F Jebbink, Martin Deijs, Marta Canuti, Salmaan Sharif, Michel de Vries, Adnan Khurshid, Tariq Mahmood, Lia van der Hoek, Syed Sohail Zahoor Zaidi

**Affiliations:** Department of Virology, National Institute of Health, Chak Shahzad, Park Road, Islamabad, 45500 Pakistan; Department of Biotechnology, Faculty of Biological Sciences, Quaid-i-Azam University, Islamabad, 45320 Pakistan; Department of Medical Microbiology, Laboratory of Experimental Virology, Academic Medical Center, University of Amsterdam, Amsterdam, The Netherlands; CBS-KNAW Fungal Biodiversity Center, Utrecht, The Netherlands; Department of Plant Sciences, Faculty of Biological Sciences, Quaid-i-Azam University, Islamabad, 45320 Pakistan

**Keywords:** VIDISCA, cDNA-AFLP, Enterovirus, Human parechovirus, Human astrovirus, Tetnovirus, Sequence independent method, Acute flaccid paralysis, Pakistan

## Abstract

**Background:**

The use of sequence independent methods combined with next generation sequencing for identification purposes in clinical samples appears promising and exciting results have been achieved to understand unexplained infections. One sequence independent method, Virus Discovery based on cDNA Amplified Fragment Length Polymorphism (VIDISCA) is capable of identifying viruses that would have remained unidentified in standard diagnostics or cell cultures.

**Methods:**

VIDISCA is normally combined with next generation sequencing, however, we set up a simplified VIDISCA which can be used in case next generation sequencing is not possible. Stool samples of 10 patients with unexplained acute flaccid paralysis showing cytopathic effect in rhabdomyosarcoma cells and/or mouse cells were used to test the efficiency of this method. To further characterize the viruses, VIDISCA-positive samples were amplified and sequenced with gene specific primers.

**Results:**

Simplified VIDISCA detected seven viruses (70%) and the proportion of eukaryotic viral sequences from each sample ranged from 8.3 to 45.8%. Human enterovirus EV-B97, EV-B100, echovirus-9 and echovirus-21, human parechovirus type-3, human astrovirus probably a type-3/5 recombinant, and tetnovirus-1 were identified. Phylogenetic analysis based on the VP1 region demonstrated that the human enteroviruses are more divergent isolates circulating in the community.

**Conclusion:**

Our data support that a simplified VIDISCA protocol can efficiently identify unrecognized viruses grown in cell culture with low cost, limited time without need of advanced technical expertise. Also complex data interpretation is avoided thus the method can be used as a powerful diagnostic tool in limited resources. Redesigning the routine diagnostics might lead to additional detection of previously undiagnosed viruses in clinical samples of patients.

**Electronic supplementary material:**

The online version of this article (doi:10.1186/1743-422X-11-146) contains supplementary material, which is available to authorized users.

## Background

The identification and characterization of viruses in clinical samples is an essential component of community health monitoring systems. A diverse range of conventional and molecular based diagnostic assays are widely used to detect these pathogens. Conventional assays like electron microscopy, cell culture and immunological methods have been successfully used for identification of viruses but in many occasions these methods failed to detect the etiological agent in clinical samples due to poor sensitivity and cross reactivity. Therefore, molecular assays like polymerase chain reaction (PCR) [1], universal primer PCR [2–4], pan viral microarray, cDNA library immunoscreening [5], substitution hybridization [6–8] have been used for detection of unknown viruses, but these methods were not always proven useful due to increased diversity of viral genome, low viral loads in clinical samples and presence of a variety of organisms in a single specimen. Therefore, there is a need to develop improved and economical diagnostic tools to deal with such problems.

In recent years the technological innovations involving sequence independent amplification techniques and next generation sequencing (NGS) have boosted virus discovery and these NGS-based techniques are becoming the standard for the discovery of viral pathogens in clinical samples. Among these innovations, sequence independent methods have been used efficiently to identify unknown viruses in diagnostic virology. One of these methods, Virus Discovery based on cDNA Amplified Fragment Length Polymorphism (VIDISCA), has proven to be a successful tool for identification of unknown viruses [9]. This method is capable of detecting both DNA and RNA viruses without prior knowledge of the viral family, and is based on restriction enzyme digestion. Nowadays VIDISCA is combined with next generation sequencing, but at the time it was developed (2004), it has been successfully used to discover novel viruses from cell culture like human coronavirus NL63 (HCoV-NL63) [10], human parechovirus type 5 and 6 [11] and human parechovirus type 1 variant [12]. Standard VIDISCA uses a two step PCR amplification protocol with a complex second selective amplification round, and subsequent isolation of PCR fragments which are not in uninfected cultures. This last amplification step is complex and labor intensive, but needed to distinguish background ribosomal RNA PCR fragments from the viral PCR products. However, pre-treatment of samples prior to VIDISCA has significantly been optimized and amplification of ribosomal RNA has diminished dramatically [13]. Therefore, a simplified VIDISCA protocol was developed, which lacks the last amplification round, and evaluated using viruses that were cultured from stool samples of acute flaccid paralysis children, and which had remained unrecognized on both cell culture and enterovirus specific real-time PCR.

## Results

**Figure 1:**
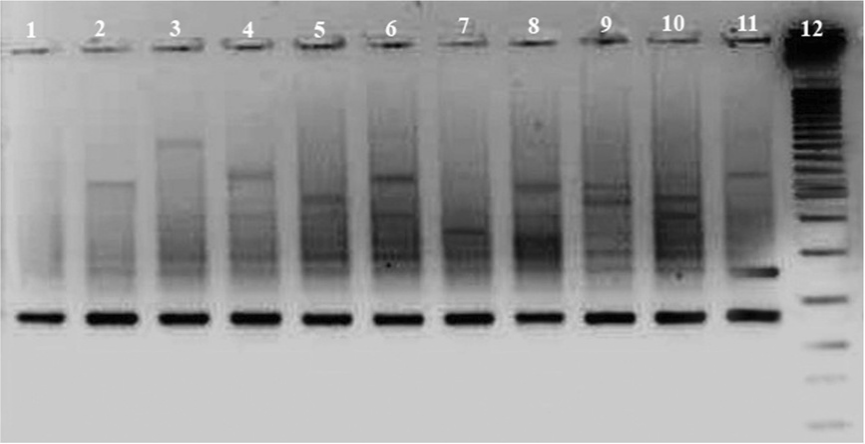
**VIDISCA PCR fragments visualized on a 3% metaphor gel which were generated after amplification in a single round PCR of 40 cycles.** Lane 1: control supernatant from uninfected RD cells; Lane 2–6: PCR product of cultured viruses harvested from L20B cells; Lane 7–11: PCR product of cultured viruses harvested from RD cells; Lane 12: 25 base pair molecular weight marker.

Virus discovery techniques are often a combination of unbiased amplification and high throughput sequencing. However, high throughput sequencing is almost impossible in developing countries like Pakistan because its cost is prohibitive. Therefore, we used a simple and cheap alternative virus discovery tool, which is a combination of a short virus culture together with a simplified version of VIDISCA (an overview of the adaptations is shown in Additional file 1: Table S1). Ten supernatants from CPE-showing cultures of stool from patients with Acute Flaccid Paralysis were tested (patient characteristics are shown in Table 1). The virus cultures had been examined by the standard diagnostics but remained negative (data not shown). All of the study samples yielded PCR fragments on 3% metaphor gel (Figure 1). Of each sample the PCR products which were present in the samples but not in the cell culture control were cut from gel and cloned in *E.coli*. Twelve to 24 cloned products were Sanger sequenced. A significant amount of nucleotide sequences showed identity to known viruses in 7 samples (70%). These viral sequences matched with members belonging to different families e.g. *Picornaviridae*, *Astroviridae* (Table 2). The proportion of eukaryotic viral sequences in each sample varied and ranged from 8.3% to 45.8%. Four human enteroviruses were identified (serotypes EV-B97, EV-B100, echovirus (E)-9 and E-21), human parechovirus type 3 (HPeV-3), tetnovirus-1 (TNV-1), and one human astrovirus (several fragments of which some with identity to type 3 (HAstV-3) and other fragments - at different locations of the genome - showing identity with human astrovirus type 5 (HAstV-5). To further characterize the viruses, the 7 VIDISCA-positive samples were amplified and sequenced with gene specific primers. For the human enteroviruses and the parechovirus the VP1 gene was used, and the ORF1a and ORF2 genes were used for the astrovirus.

**Figure 2:**
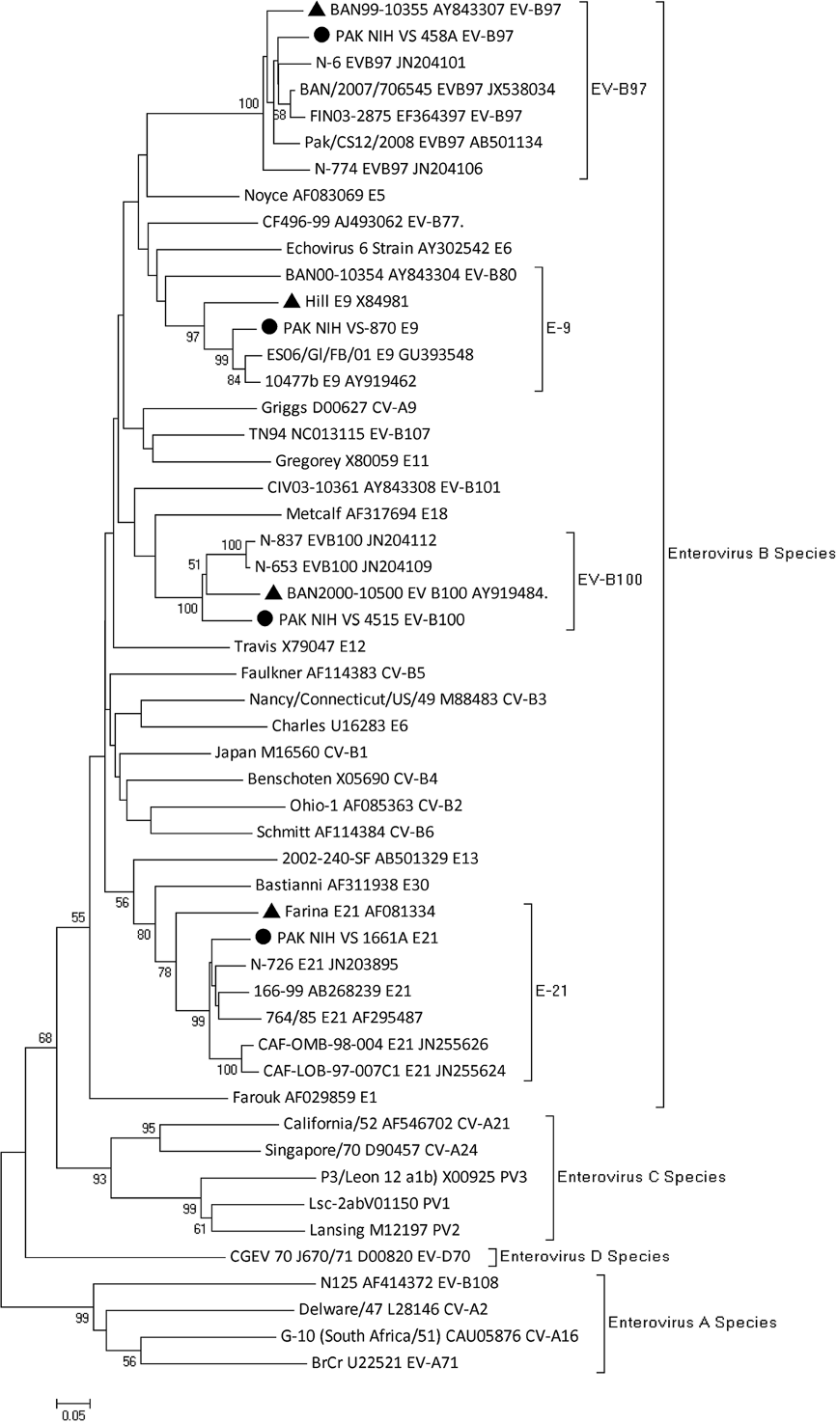
**Phylogenetic analyses based on VP1 nucleotide sequences of PAK NIH isolates and representative sequences of enterovirus serotypes retrieved from GenBank.** The isolates characterized by VIDISCA are represented by a ‘●’ taxon marker. Prototype strains are labeled with taxon markers ‘▲’. The tree was constructed using neighbor-joining (NJ) method and Kimura 2-Parameter (K2P) model in MEGA 4.0 and evaluated with 1000 bootstrap pseudoreplicates. Bootstrap values greater than 50 are indicated at the respective nodes and the scale bar represents the evolutionary distance.

**Figure 3:**
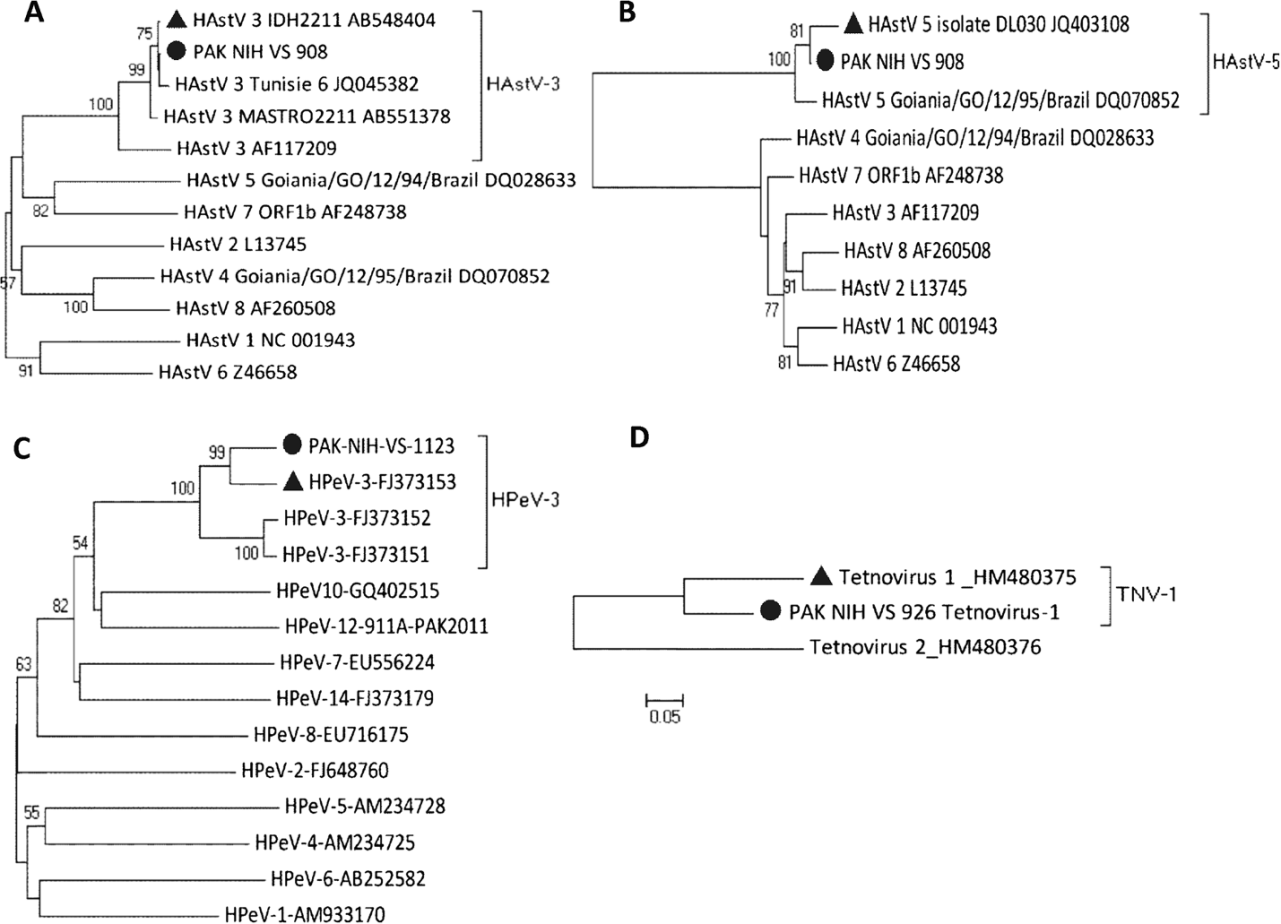
**Phylogenetic analyses of A) Human astrovirus ORF2 B) Human astrovirus ORF1a C) Human Parechovirus VP1 and D) Tetnovirus unknown gene.** The representative sequences of the serotypes are retrieved from GenBank (accession numbers are included in the virus names). The isolates from this study are represented by a ‘●’ taxon marker. The closely related strains are labeled with taxon markers ‘▲’. The tree was constructed using neighbor-joining (NJ) method and Kimura 2-Parameter (K2P) model in MEGA 4.0 and evaluated with 1000 bootstrap pseudoreplicates. Bootstrap values greater than 50 are indicated at the respective nodes and the scale bar represents the evolutionary distance.

Phylogenetic analyses of the VP1 gene were performed to investigate the genetic relationships between the human enterovirus strains from this study (PAK-NIH VS458A, VS870, VS1661A and VS4515) and the enterovirus serotypes in GenBank (Figure 2). The nucleotide identity between the study strains and their reference prototypes ranged from 78.7% to 91.9% and within each serotype from 79.2% to 98.8%. The nucleotide sequence identity between PAK-NIH-VS1661A and the closest relative echovirus 21 (Farina;AF081334) was only 78.7% and thus this isolate represents a separate genotype, matching most closely with strains from India, China and Sweden (Genbank accession numbers are shown in Figure 2). Phylogenetic analysis of the VP1 coding region of HPeV strains available in GenBank and our study isolate (PAK-NIH-VS1123) showed clustering with HPeV type 3 strain 651689 (FJ373153) isolated in Amsterdam, the Netherlands (Figure 3). The strains had 92% nucleotide identity and 99% amino acid identity with each other.

The ORF1a and ORF2 sequences of astrovirus PAK-NIH-VS908 were aligned and compared with sequences from GenBank including those who gave the largest similarity in blast alignments. The genetic analysis in ORF1a gene (Serine protease) showed that the study isolate belongs to human astrovirus type 5 representing 94.5% nucleotide identity and 99% amino acid identity with HAstV isolate DL030 (JQ403108). On the other hand, genetic analysis in ORF2 gene (capsid protein) showed that PAK-NIH-VS908 shared 99.7% nucleotide and 100% amino acid similarities with HAstV type 3 isolate IDH2211 (AB54844), a finding which matches with the results from VIDISCA and we conclude that the astrovirus is a recombinant, with a recombination site between ORF1a and ORF2. Phylogenetic trees were constructed based on sequences of both genes separately which showed that PAK-NIH-VS908 has different grouping patterns in relation to the reference HAstV prototypes (Figure 3), confirming that the isolate is most probably a recombinant (Figure 3).

The partial nucleotide sequence of PAK-NIH-VS926 (222-nucleotides) showed most identity with tetnovirus-1 strain (HM480375) isolated from an AFP patient in Afghanistan (Figure 3). Pairwise distance calculation showed 82.9% nucleotide identity with its closest match and therefore PAK-NIH-VS926 probably presents a divergent isolate within this group.

**Table 1:** **Clinical and demographical data of children with acute flaccid paralysis in this study**

S. NO.	Sample ID	Collection year	Male (M)/Female (F)	Age (months)	Asymmetrical paralysis	Fever
1	PAK_NIH_VS1661A	2010	M	25	Yes	Yes
2	PAK_NIH_VS458A	2009	M	09	Yes	Yes
3	PAK_NIH_VS870	2009	M	14	Yes	Yes
4	PAK_NIH_VS4515A	2010	M	48	Yes	Yes
5	PAK_NIH_VS908	2008	F	06	No	Yes
6	PAK_NIH_VS926	2008	M	NA	Yes	Yes
7	PAK_NIH_VS1123	2009	F	51	Yes	No
8	PAK_NIH_VS242	2009	M	25	No	Yes
9	PAK_NIH_VS3099	2009	F	70	No	No
10	PAK_NIH_VS7113	2008	F	11	Yes	No

**Table 2:** **Identification of viruses in AFP patients by VIDISCA**

S. NO.	Sample ID	No of colonies sequenced	No of viral sequences	% of viral sequences	VIDISCA-result
**1**	PAK_NIH_VS1661A	24	2	8.3	Echovirus −21
**2**	PAK_NIH_VS458A	24	7	29.1	Enteovirus-B97
**3**	PAK_NIH_VS870	24	8	33.3	Echovirus-9
**4**	PAK_NIH_VS4515A	24	10	41.6	Enterovirus-B100
**5**	PAK_NIH_VS908	24	11	45.8	Human astrovirus type 3 and type 5
**6**	PAK_NIH_VS926	12	1	8.3	Tetnovirus-1
**7**	PAK_NIH_VS1123	12	1	8.3	Human Parechvirus type 3
**8**	PAK_NIH_VS242	12	0	0	-
**9**	PAK_NIH_VS3099	12	0	0	-
**10**	PAK_NIH_VS7113	24	0	0	-

## Discussion

In recent years, sequence independent PCR approaches have revolutionized identification of unknown and novel viruses either alone or in combination with conventional methods. In this study a simplified version of VIDISCA was used to detect human enteroviruses, a human parechovirus type 3, a human astrovirus, and a tetnovirus-1 in cell culture. Our data show that a wide range of distinct viruses that remained unrevealed through routine assays can be identified in a relatively short amount of time with an easy to use method. The VIDISCA method is based on cDNA-AFLP and one characteristic of this method is that it uses a double PCR strategy, with in the second round of amplification selective primers (selective PCR-round). These selective primers are extended at the 3′ site with one or two nucleotides, and in the selective PCR various combinations of primers are used in order to amplify everything which was amplified in the first PCR. This selective PCR step is a complex and laborious step in VIDISCA, but was needed previously to distinguish viral PCR fragments from ribosomal RNA amplicons. However, an improved purification and reverse transcription step which strongly diminishes ribosomal RNA amplification has recently been published, and therefore the laborious selective amplification step might not be needed [13]. Here, we show that in the majority of virus cultures simplified-VIDISCA can quickly reveal the infecting agent. We previously published that VIDISCA in its original setting can identify a virus in picornavirus-cultures in >90% of the cultures [11]. A sensitivity of 70% which we have here is largely comparable, but we must mention here that we did not compare the sensitivity of traditional VIDISCA and simplified VIDISCA directly in our study.

The possible failure of screening of study samples via routine assays may be due to increased genetic diversity at PCR priming annealing sites [14, 15]. Seven viruses (EV-B97, EV-B100, E-9 and E-21, HPeV-3, HAstV-3/5 and TNV-1) were identified which originate from stool samples of AFP patients. The clinical significance of these viruses has great impact on public health and constitutes a health risk for the community. All isolated enteroviruses belong to enterovirus B species containing the most frequently isolated serotypes that more commonly cause meningitis, myocarditis and neurological disorders [16–20]. Some enteroviruses cause severe and potentially life-threatening illness and there is currently no antiviral treatment available for enterovirus infection [19]. Phylogenetic clustering of our study isolates reveals that the enteroviruses are related to the circulating strains which have been reported in neighboring countries: India and Bangladesh. Our analysis also showed that the nucleotide sequence of echovirus 21 isolate (PAK-NIH VS1661A) has low nucleotide identity (78.7%) with prototype Farina strain (AF081334) that fulfils the criterion for a genotype [21] and therefore we suggested that it is a separate genotype of E-21 circulating in the area. Importantly, the emergence of new genetic lineages of enteroviruses in the community is an alarming situation for Pakistan where there is no enterovirus surveillance system. Aside from poliovirus, which is the target pathogen of the polio eradication strategy, the non-polio enterovirus detection in the laboratory is only a “part-outcome” of AFP surveillance. In Pakistan, despite the significant number of isolated non-polio enteroviruses, limited information is available with regard to its incidence, diversity and circulation pattern. Consequently, it is the right time to prepare for future tasks and to give attention towards non polio viruses causing AFP which is an equal cause of concern while we are approaching towards the polio eradication era.

Similarly, HPeVs are known to cause a variety of clinical symptoms similar to enteroviruses like gastroenteritis and occasionally flaccid paralysis and encephalitis [22, 23] particularly in infants, and they are considered a major cause of infant mortality worldwide. HPeV types 1, 5, 6, 7 and 12 were isolated from stool samples of non polio AFP patients <3 years of age [24–26]. In this study we identified HPeV-3 in a seven months old paralytic infant having fever. Our findings was in agreement with previous studies in which HPeV-3 was isolated from children aged less than three years having transient paralysis and sepsis like illness [22, 27].

Astrovirus is one of the known causes of acute gastroenteritis in humans, mostly in young children. It has been isolated earlier from stool of an AFP patient [28]. In this study, an astrovirus was isolated from a six months old child. A follow up examination after 60 days indicated the complete recovery of patient from paralysis. Phylogenetic analyses of PAK-NIH-VS908 based on ORF1a and ORF2 genes clustered this isolate in two different genotypes; HAstV-5 and HAstV-3. This genotype discrepancy among phylogenetic analysis of both genes most likely represents a possible recombination event in the evolution of HAstVs. Recombination is a normal phenomenon among these viruses [29–32].

PAK-NIH-VS926 is the most divergent strain of the type 1 tetnoviruses. Tetnovirus-1 is an RNA virus containing two large ORFs encoding structural and non-structural proteins. The non-structural protein of tetnovirus-1 contains an RNA dependent RNA polymerase (RdRp) and a cysteine-like protease domain. The genomic organization of TNV-1 is more closely related to the viruses classified in the family *Tetraviridae.* Tetnoviruses are closely related to nodaviruses within the RdRp protein. The host of nodaviruses is fish and arthropods, including insects. Tetnovirus-1 and −2 have been isolated earlier from stool samples of AFP children of Afghanistan [33]. Future studies are needed to identify the origins of these viruses and to clarify their in vitro replication and pathogenic potential in AFP children.

Nowadays sequence independent methods followed by high throughput sequencing [34–36] are becoming more promising means for detection of novel pathogens. However, highly developed technical skills, involvement of bioinformatics’ support, adequate computing resources, advanced data interpretation and high costs are major barriers for their use in developing and resource-limited countries, which are exactly those regions where outbreaks of new pathogens are likely to occur [28, 37–39]. Therefore, we consider the adaptation of VIDISCA a most appropriate method for resource poor countries and it can be successfully completed in a limited time without technical difficulties. This assay is applicable independently in any laboratory having only PCR and sequencing facilities and can be reproduced easily from the literature.

## Conclusion

Our data support that VIDISCA may be of great utility for the identification of viruses that escaped conventional diagnostics. Furthermore, avoiding the cost, time, labor and use of specialized equipments made it an efficient and powerful diagnostic tool for resource poor countries. Finally, our findings also provide confidence to improve and redesign the routine diagnostic assays which might lead to additional detection of previously undiagnosed viruses in clinical samples of patients.

## Methods

### Patient samples and virus culture

A total of ten stool samples from AFP children aged less than 15 years were selected from the sample bank of acute flaccid paralysis patients at the Virology department, National Institute of Health, Pakistan (Table 1). All these samples showed a cytopathic effect (CPE) in rhabdomyasarcoma cells (RD) and mouse cells that have receptors for human polioviruses (L20B) and found negative for enterovirus by real-time reverse transcription PCR targeting the 5′UTR of the genome [40]. Cell culture controls each for L20B and RD cells were included

### Ethics statement

This study was approved by the Internal Review Board of National Institute of Health, Pakistan. Written informed consent was obtained from parents (or guardians) of participating patients.

### VIDISCA

The pretreatment of samples was used prior to VIDISCA to reduce background nucleic acids. Samples were centrifuged (10.000 g) and supernatant was digested with Trubo DNase (Ambion). Extraction and isolation of nucleic acids was performed according to the protocol of Boom et al. [41]. Reverse transcription was performed using random hexameres, designed such that they do not anneal to ribosomal RNA as described by Endoh et al. [42], and Klenow polymerase was used for second strand synthesis (New England Biolabs). The double stranded DNA was subsequently digested and annealed to adaptors as described by de Vries et al. [13], followed by PCR, only one round of 40 cycles, which is different from the protocol as described [10] A cookbook version of the method is presented as Additional file 2: (Doc. S2). Sequences were trimmed from raw sequencing data using Sequencher programe version 4.9 (Gene Codes corporation) and analyzed online (http://www.ncbi.nlm.nih.gov/BLAST). The blastn and blastx algorithms were used to identify sequences with similarity to known viruses in GenBank and those sequences identified as viral were further classified into viral families based on the taxonomy of the best hit.

### Typing and viral characterization

All samples with sequence identity to known viruses were characterized with gene specific PCRs to confirm the presence of the pathogen in the samples. The capsid-encoding VP1 gene of human enteroviruses and human parechovirus type 1 was amplified by using primers 490/492, 491/493 [43] and VP1-parEchoF1/VP1-parEchoR1 [44] respectively. Similarly ORF1a and ORF2 genes of human astrovirus were amplified by using primers Mon269/Mon270 and Mon340/Mon348 [29] respectively. Amplified DNA products were visualized on 2% agarose gel and sequenced with the same primers as used in PCR. Phylogenetic analyses were conducted using MEGA (Molecular Evolutionary Genetic Analysis) version 4.0 [45] and p distances (nucleotide and amino acids) were computed. Trees were constructed by Neighbor Joining (NJ) method using Kimura 2-parameter (K2P) model for nucleotide sequences [46] and bootstrap values with 1000 pseudo replicate data sets were estimated. Sequences from this study were submitted to GenBank with the Accession numbers KF453625-KF453632.

## Electronic supplementary material

Additional file 1: Table S1: An overview of the adaptations in the VIDISCA protocol. (DOC 37 KB)

Additional file 2: Doc. S2: A cookbook version of the simplified VIDISCA method. (DOC 93 KB)
