# Supplementary material for: Identification and characterization of unrecognized viruses in stool samples of non-polio acute flaccid paralysis children by simplified VIDISCA
Source: Virol J. 2014 Aug 12;11:146. doi: 10.1186/1743-422X-11-146 (PMC4254409; doi:10.1186/1743-422X-11-146)
Supplement: Supplementary file 1 — Additional file 1: Table S1: An overview of the adaptations in the VIDISCA protocol. (DOC 37 KB) [file 12985_2014_2473_MOESM1_ESM.doc]

**Table S1: An overview of the Adaptations in the VIDISCA Protocol**

|  | **VIDISCA DIFFERENTATION** | | |
| --- | --- | --- | --- |
| **Procedure** | **Standard VIDISCA** | **Simplified VIDISCA** | **VIDISCA 454** |
| **Culture of a virus** | YES | YES | NO |
| **Removal of cells/mitochondria** | YES | YES | YES |
| **DNase treatment** | YES | YES | YES |
| **Nucleic acid isolation** | YES | YES | YES |
| **Reverse transcription** | YES | YES | YES |
| **Second strand synthesis** | YES | YES | YES |
| **Restriction enzyme digestion** | MseI and Hinp1-I | MseI | MseI |
| **Ligation to anchors** | YES | YES | YES |
| **Amplification by PCR with anchor specific oligonucleotides** | 35 cycles | 40 cycles | 28 cycles |
| **Second round of PCR-amplification with combinations of selective oligonucleotides** | YES | NO | NO |
| **Selection of specific fragments via metaphor agarose gel electrophoresis (S)(V)** | YES | YES | NO |
| **Clonal amplification by** | TA-cloning | TA-cloning | Emulsion PCR |
| **Sequencing by** | Sanger sequencing of the plasmid inserts of 12 to 24 transfected colonies | Sanger sequencing of the plasmid inserts of 12 to 24 transfected colonies | Next Generation Sequencing |
