# Supplementary material for: Identification and characterization of unrecognized viruses in stool samples of non-polio acute flaccid paralysis children by simplified VIDISCA
Source: Virol J. 2014 Aug 12;11:146. doi: 10.1186/1743-422X-11-146 (PMC4254409; doi:10.1186/1743-422X-11-146)
Supplement: Supplementary file 2 — Additional file 2: Doc. S2: A cookbook version of the simplified VIDISCA method. (DOC 93 KB) [file 12985_2014_2473_MOESM2_ESM.doc]

**Doc. S2: Cookbook Versioin of the Simplified VIDISCA Method:**

Start with a clinical specimen/virus culture supernatant suspected to contain viruses of interest.

**Pre-treatment**

- Centrifuge 110 μl sample for 10 minutes, 10.000 *g*
- Transfer 100 μl supernatant into new Eppendorf tube, take care not to include the pellet/debris

DNAse treatment:

- Add 10 μl Turbo DNase (2U/μl; Ambion), 12 μl 10 X DNase buffer (Ambion) to the 100 μl supernatant
- Incubate 30 minutes at 37 ºC

## Isolation^¶^ of RNA and DNA ^2^

- Add 900 μl L6, mix well and incubate 10 minutes room temperature
- Add 40 μl of silica suspension, incubate 10 minutes room temperature with continuous shaking
- Spin down with maximum speed (30sec).
- Take off the supernatant.

Wash the silica-pellet:

- Add 900 µl L2 buffer, vortex and centrifuge at max. speed for 30 sec.
- Take off the supernatant.
- Repeat the L2 washing step.
- Add 900 µl 70% EtOH, vortex and centrifuge at max. speed for 30 sec.
- Take off the supernatant.
- Repeat the 70% EtOH washing step.
- Take off the supernatant.
- Add 900 µl acetone, vortex and centrifuge at max. speed for 30 sec.
- Take off the supernatant.
- Dry the silica for 5 minutes at 56 ºC.

Elution of nucleic acids:

- Add 40μl sterile water*^¶^* + 10 µl mixed rRNA-blocking-oligo’s (4 µM each) [rRNA-blocking-oligo sequences are included at the end of this protocol]
- Incubate 10 minutes at 56 ºC, with continuous shaking
- Spin down at maximum speed for 2 minutes
- Gently transfer ~40 µl eluate into a new tube without taking any silica particles

**^¶^ Remark: If you are using a commercial kit for isolation of nucleic acids, add the rRNA-blocking oligo’s in the final elution step.**

**RT reaction**

- Prepare the RT mixes:
- RT-mix I
- 2.5 μl non-ribosomal hexamers 1 μg/μl ^3^
- [hexamer sequences: see end of this protocol]
- 3 μl 10X *E. coli* ligase buffer (Invitrogen)
- 2.4 μl MgCl_2_ (100mM)
- 2.1 μl water (sterile)
- RT-mix II
  - 2 μl 10X *E. coli* ligase buffer, (Invitrogen)
  - 1 μl SuperScript II (200U/μl Invitrogen)
  - 0.8 μl dNTPs (25mM of each)
  - 15.2 μl water (sterile)
  - 1 µl DTT (0.1 M)
- Centrifuge the eluate from the isolation step for 30s with maximum speed (to remove any residual silica)
- To 20 µl of the eluate add 10 µl of RT-mix I. Incubate for 2 minutes at room temperature.
- Add 20 µl of RT-mix II.
- Incubate for 90 minutes at 37 ºC, followed by 20 minutes at 70 ºC.

### Second strand synthesis

- Add 100 μl of second strand synthesis mix:
- 10 µl NEB2 10X buffer
- 1 µl Klenow polymerase (3' - 5’ exo^-^) (5U/μl; New England Biolabs)
- 1.5 µl RNAseH (5U/μl) (New England Biolabs)
- 1 µl dNTPs (25mM each)
- 86.5 µl sterile water
- Incubate at 37 ºC for 90 minutes (total volume 150 μl)

**Phenol/chloroform extraction and ethanol precipitation**

- Mix the sample with 150 µl phenol/chloroform/iso-amylalcohol mixture (Invitrogen Ultra-pure PCI, 25:24:1 v/v) and vortex vigorously
- Spin down the sample 1 min. at max. speed and transfer 140 µl upper layer (water phase) to a new Eppendorf tube
- Add 350 µl of 100% ethanol (2.5 volume) and 14 µl (0.1 volume) of 3M sodium acetate (pH 5.2) and vortex.
- Precipitate the nucleic acids overnight at -20 ºC
- Spin down the sample for 25 minutes (max. speed; at least 7500*g*) at +4 ºC
- Discard supernatant
- Add 200 μl of (fresh) 70% ethanol
- Centrifuge for 25 minutes (max. speed; at least 7500*g*) at +4 ºC
- Remove ethanol and air-dry the pellet for 15 minutes at room temperature
- Dissolve the pellet in 30 μl sterile water

**Digestion of ds cDNA with Mse I restriction enzyme**

- Prepare digestion mix:
- 4 μl 10X buffer (New England BioLabs, supplied with Mse1)
- 5 μl water **(**sterile)
- 1 μl Mse1 restriction enzyme **(**10U/μl; New England Biolabs)
- Add 10μl of the digestion mix and incubate 2 hrs at 37 ºC,
- Continue with ligation (no storage step allowed at this point, since Mse1 needs to be active during ligation)

**Ligation of adaptors to the digested fragments**

Prepare ligation mix:

- 1 μl MID1-A adaptor (5 µM, see below)
- 1 μl B adaptor (5 µM, see below))
- 2 μl 5x ligation buffer (Invitrogen)
- 1 μl T4 ligase **(**5U/μl; Invitrogen)
- 10 μl sterile water
- Add the ligation mix (15μl) to the digested sample (40 μl).
- Incubate 2 hrs at room temperature

#### Construction of A and B adaptors

Mix:

- 25 μl Top oligo (MID1-TopA or Top-B) (20 µM)
- 25 μl Bottom oligo (MID1-bottomA or Bottom-B) (20 µM)
- 5 μl 5X ligation buffer (Invitrogen)
- 45 μl sterile water

Heat to 65 ºC for 5 minutes and cool down slowly to room temperature.

Store at -20 ºC

**PCR reaction**

- Transfer 10 μl of the ligated mix into a PCR tube and mix with 40 μl of PCR mix:
- 31.25 μl sterile water
- 0.75 μl MgCl_2_ (100mM)
- 5 μl 10x PCR buffer (Roche)
- 0.5 μl dNTPs (25mM of each)
- 1 μl Tit-PCR-A (20 µM)
- 1 μl Tit-PCR-B (20 µM)
- 0.5 μl AmpliTaq polymerase (5U/ul; Roche)

Perform a PCR reaction according to the following profile:

5min 95 ºC

1min 95 ºC|

1min 55 ºC| 40 cycles

2min 72 ºC|

10min 72 ºC

10min 4 ºC

Store PCR products overnight at -20 ºC, or continue with gel analysis.

**Agarose gel analysis**

- Mix 15 µl of PCR product and 5 µl of loading dye and run the DNA on a 3 % Metaphor agarose gel (Cambrex), or, if Metaphor agarose is not available, 1.5% agarose gel. Use TBE running buffer, and ethidium bromide staining.
- Cut from gel the fragments of interests (those that are not in the negative control), purify the DNA and clone into a cloning vector (for example via TA cloning (Invitrogen)
- Sequence the inserts of 12 to 24 colonies via Sanger sequencing, use primers annealin to the cloning vector (e.g. Sp6 or T7)

**Oligonucleotide sequences VIDISCA**

**Adaptor oligonucleotides (HPLC purified)**

- MID1-top-A GCCTCCCTCICGCCATCAGACGAGTGCGTA
- MID1-bottom-A TATACGCACTCGTCTGATGGCGCGAGGGAGGC
- Top-B GCCTTGCCAGCCCGCTCAGA
- Bottom-B TATCTGAGCGGGCTGGCAAGGC

**PCR oligonucleotides**

- Tit-PCR-A CGTATCGCCTCCCTCGCGCCATCAG
- Tit-PCR-B CTATGCGCCTTGCCAGCCCGCTCAG

**rRNA-blocking oligonucleotides ^4^**

- 1-Morrna 5’ CTTTCGCTCTGGTCCGT 3’ –C6 [18S, nt. 977 – 1071]
- 2-Morrna 5’ CACTAATTAGATGACGAGG 3’–C6 [28S, nt. 3767 – 3785]
- 3-Morrna 5’ TGACATTCAGAGCACTGG 3’–C6 [28S, nt. 3679- 3696]
- 4-Morrna 5’ GTTACTGAGGGAATCCTG 3’ –C6 [28S, nt. 72 – 89]
- 5-Morrna 5’ CACCAGTTCTAAGTCGG 3’–C6 [28S, nt. 3580 – 3596]

**Non-ribosomal hexamers ^3^**

References

^1^ van der Hoek L *et al*. "Identification of a new human coronavirus" Nat Med. 2004, Apr;10(4):368-73.

^2^ Boom*, et al.*, "Rapid and simple method for purification of nucleic acids," J Clin Microbiol. 28(3), 495 (1990).

^3^  Endoh*, et al.*, "Species-independent detection of RNA virus by representational difference analysis using non-ribosomal hexanucleotides for reverse transcription," Nucleic Acids Res 33(6), e65 (2005).

^4^ de Vries *et al.* "A sensitive assay for virus discovery in respiratory clinical samples"

PLoS ONE 6(1): e16118. doi:10.1371/journal.pone.0016118 (2011)
